# Diffuse Reflectance Spectroscopy; Applications, Standards, and Calibration (With Special Reference to Chromatography)

**DOI:** 10.6028/jres.080A.055

**Published:** 1976-08-01

**Authors:** R. W. Frei

**Affiliations:** Analytical Research and Development, Pharmaceutical Department Sandoz Ltd., 4002 Basel, Switzerland

**Keywords:** Chromatography, color matching, color measurement, diffuse reflectance, Kubelka-Munk function, reflectance, reflectance standards, thin layer chromatography

## Abstract

The multitude of areas in which diffuse reflectance spectroscopy can be applied has been described in several books and reviews and ranges from color measurements of textiles, pharmaceuticals, building materials, paper and pulp materials etc., to adsorption studies and other basic investigations in physical, inorganic and organic chemistry.

The major area of application is still the measurement of color which has become indispensible in the quality control of colored products, dyes and pigments. Color matching practices and techniques with sophisticated instrumentation which can be fully computerized as well as the use of simpler filter instruments for quality control are mentioned.

Transferability of reflectance data i.e., color coordinates, depends on the quality of standards particularly when absolute measurements are desired. The difficulty of finding suitable “white standards” with good reflection properties at low UV and with a good long term stability is discussed. Similar arguments hold for sphere coating materials. For the measurement of fluorescing surfaces suitable standards are lacking which renders transfer of such data almost impossible.

The usefulness of diffuse reflectance techniques to study adsorption phenomena on small particle adsorbents is demonstrated with a malachite green-*o*-carboxylic acid lactone system studied by Kortüm. This or similar systems could be adopted to the measurement of relative surface areas on certain chromatographic adsorbents yielding more realistic values than the BET-method.

The most recent area of application has been in the field of chromatography for the in situ evaluation of chromatographic zones in flat-bed chromatography, electrophoresis and isoelectric focusing.

In chromatography, standardization is less problematic since usually relative measurements are sufficient. On the other hand one has to find suitable calibration procedures. The use of the Kubelka-Munk function is often questionable since we are usually not dealing with layers of infinite thickness and below 300 nm the conventional adsorbents such as silica gel, alumina or cellulose are strongly absorbing. Experiences with a new function combining the laws of Kubelka-Munk and Lambert-Beer are therefore presented.

The problem is also to find calibration techniques which account for chromatographic parameters. Until recently it was believed that a quantitative evaluation of chromatograms required a number of reference zones to be developed on the same chromatogram. In our experience this is no longer true. A novel calibration technique which utilizes the concept of transferable calibration factors is discussed. With this approach a quantitative evaluation of a chromatogram with only one reference spot is possible. Here again scanning and data acquisition can be fully automated. The application of proper calibration procedures to differential reflectance techniques and the measurement of multi-component systems is briefly mentioned.

Finally it is demonstrated that it is possible to carry out in situ quantitative measurements on low UV absorbing compounds (down to 190 nm) separated on silica gel surfaces, provided suitable techniques and instrumentation are used.

## I. Introduction

Diffuse reflectance spectroscopy has found its application in a number of areas. The first and still most prominent is in the field of color measurement and color matching. The paper, paint, dye, textile, printing, and ceramics industries have made use of this technique for the measurement of color in routine quality-control functions as early as 1920. At that time the first useful filter reflectometers became available [1]^1^ and a little later a spectrophotometer type reflectometer [2] was manufactured.

A first comprehensive treatise of color measurement became available in 1936 [3]. Since that time the literature on color measurement has increased exponentially peaking out somewhat in the 1960’s with not less than seven books appearing on this subject [4–10].

Later the principles of color measurements were extended to other areas including studies of biological systems, geological specimens, food stuff, building materials, and pharmaceuticals. With the expansion and improvement of the quality of available reflectometers on the instrumental scene it also became possible to carry out physical-chemical measurements, requiring extremely reproducible experimental and instrumental conditions. Comprehensive discussions and surveys have been given on these applications and on the instrumental developments in several books [11–15] and reviews [16–17].

Essentially no new aspects have been added to this to my knowledge. I see therefore not much point in repeating information which can be read in these cited references.

For pharmaceutical applications the technique is being used extensively for production control, formulation studies and for investigations of aging and illumination effects. This has been developed to the stage of automatic tablet inlet systems to the Hunter Color Meter^2^ D25 DA.

The data aquisition is fully automated with a Hewlett-Packard 9100 desk calculator. This system will be discussed and published at an international pharmaceutical congress in Berlin in spring 1976 [18].

The field of instrumental color matching and color formulation has also strongly developed in the direction of automation and data evaluation by on-line and off-line computer systems, and I shall briefly mention this area.

The newest area of application of diffuse reflectance techniques has been in the field of chromatography [14–16, 19] for the in situ evaluation of chromatographic zones in flat-bed chromatography (PC, TLC), electrophoresis and isoelectric focusing. It is therefore not surprising that the most rapid changes and developments have taken place in this field, and I shall attempt to present some of the recent work in this area which has partially not yet been published.

Closely related to all these applications have been the problems of standardization and calibration. These, as we shall see, are still far from being solved satisfactorily and an assessment of the present status shall be attempted in this paper.

## II. Instrumental Color Measurement

The technique of color matching and formulation by modern instrument-computer combinations is in a worldwide rapid development stage. This development is catalyzed by the growing need of color consuming industries to optimize the coloration processes. Color and dye manufacturers, instrument makers, and their customers alike are therefore responsible for this recent surge in color measurement technology.

Unfortunately, very little information is available on this relatively young science and much of it is already out of date. This is not astonishing, considering that the major developments come from color manufacturing industries and competition has prevented much of the know-how to become accessible.

One of the better accounts of the present state of the art was given by Gall [20]. Other workers, such as Brockes [21] and Kuehni [22], have also reported on this subject, the former specifically on textiles. A brief but very illustrative introduction into the concept of color in general and color measurement in particular has been given by Berger and Brockes [23] which can be highly recommended to new-comers in the field.

On the instrument side one can observe a trend to integration of on-line computers into more sophisticated reflectometers which results in a reduction of software requirements for the user. The disadvantage is the complete dependence of each operation step on the computer which can make trouble shooting difficult for the non specialist. Another trend on the market of such systems is an increased emphasis and demand for stand-alone formulation systems (available also to smaller colorists) rather than for time-sharing systems.

### A. Types of Measuring Systems

Three different types of automatic color matching and formulation systems are now frequently used in larger companies in North America, Europe, Japan, and Australia.

1. The first system consists of a high-quality spectrophotometer for automatic measurement of reflectance spectra, an electronic interface for datastorage, a connection to the teletype and a large centralized computer facility.
  The spectrophotometer measures the reflectance of standard color samples (at least 6 different concentrations per dye) and of the samples to be matched. The reflectance data are transferred via the interface to the teletype and presented in a suitable form (cards or tapes).
  One then feeds these data to the computer centre along with a characterization of the samples, dyes and substrates to be used for a computation of the formulation, price and metamerism. This can be done via a cable connection or completely off line.
2. Following the trend of decentralization, instrument manufacturers are now marketing complete systems for automatic computation of formulations. They consist again of a high-quality monochromator instrument or a reflectometer equipped with filters producing spectral bands of 10 nm or 20 nm width. They then have an interface to a teletype and an on-line minicomputer. The spectral data are fed to the minicomputer and the same data as above are produced. The same system also can be utilized for corrections of formulations.
  The teletype in this case has two functions. It serves as the means to feed-in manually or automatically the additional data needed for formulation such as color codes and names of dyes and substrates, maximum number of dyes to be used per formulation (usually 3–4) etc. In addition it prints the final formulations, price, metamerism and other data. The software is often provided by the instrument manufacturer.
3. Since systems of type 2 are still too expensive to be used by a smaller coloring outfit or the individual colorist in a laboratory, a third system has been developed (by CIC). It makes use of system 1 as a centralized facility in a large company or a centralized service facility for colorists and a low-cost filter colorimeter which is placed directly in the colorist’s laboratory or production unit.

With this filter instrument the day-light color coordinates *X*, *Y*, *Z* of the sample are measured. In case of metamerism the color coordinates for an artificial illumination source have to be determined. These data are then transferred to the central facility. The spectrophotometer is then solely used to measure the dyes to be used in the formulation and the blank measurement on the substrate.

The disadvantages of this systems are time delays for the availability of the formulation and an increased risk for deviations from the true sample. This can be due to larger systematic errors on the low-cost unit and poor correlation of the measuring geometries on the two instruments.

### B. Criteria for the Evaluation of Measuring Systems

The most important factors for the efficiency of a formulation computing system are the quality of the calibration samples of the dyes available for the formulation and the quality of the software. One would expect the following information to be provided by an useful system:

1. Computation of all the possible alternatives for a formulation (combinations of 2–4 dyes) or indications for adjustments with one additional dye if none of the possible combinations leads to the desired result.
2. For each possible formulation the degree of metamerism with respect to one or more standard illuminants and compared to day-light condition should be given.
3. The color difference between sample to be matched and each of the possible formulations should be computed automatically for day-light condition.

The advantages of a color measuring system for computation of formulation as described above in comparison to the conventional use of sample collections are obvious:

1. The computation and fine-adjustment can be done faster since the chances of hitting the correct solution are enhanced.
2. An efficient system usually provides several solutions of which the optimal with regard to quality of the color, metamerism and price can be chosen (such a choice of course requires the services of a specialist).
3. The system is usually of sufficient flexibility to be used for trouble shooting in coloring processes (i.e., identification and quantitation of sources of error).

The systematic use of such formulation-computation systems will therefore result in a gradual upgrading of coloring techniques at lower prices.

## III. Standardization

In the majority of cases for work carried out within one laboratory measurements relative to a reflection standard tailored to a particular experiment are sufficient. For adsorbate-adsorbent interaction studies for example, one would choose the same adsorbent as a reference material. The same holds for chromatographic work. Instruments are then utilized either in the substitution mode or the comparison mode (double beam operation). The level of instrument technology is now sufficiently good to assure a satisfactory reproducibility of measurements on the same instrument [14]. The problem arises when data have to be reproduced by several different laboratories. Instrument and measurement geometries are far from being uniform, which causes considerable variations in measured values. The trend for adaptation of a uniform integration sphere design which would permit at least the reproduction of reflectometer values with instruments of different manufacturers can now be observed. The inherent limitation of construction possibilities with such a standard sphere, however, still keeps many instrument designers reluctant to follow this trend.

But even this would not guarantee the availability of absolute reflectance values, independent of the instrumentation, since such a condition depends strongly on the quality of available standards. W. Erb [24] has recently discussed the current status of reflection standards and it is apparent that little progress has been made during the past years to obtain better standard materials.

The properties that one would expect from a useful standard are as follows:

— They should be rugged enough for transportation or at least easily reproducible in each laboratory
— Along with this goes the demand for easy handling and a minimum of contamination danger.
— They should be relatively inert and stable toward radiation and temperature fluctuations and have good aging properties.
— Other requirements include an ideally diffuse reflecting, homogenous and smooth surface which is nontransparent, nonfluorescent and spectrally nonselective.

Obviously a reference material having all these properties does not exist.

**Figure 1:**
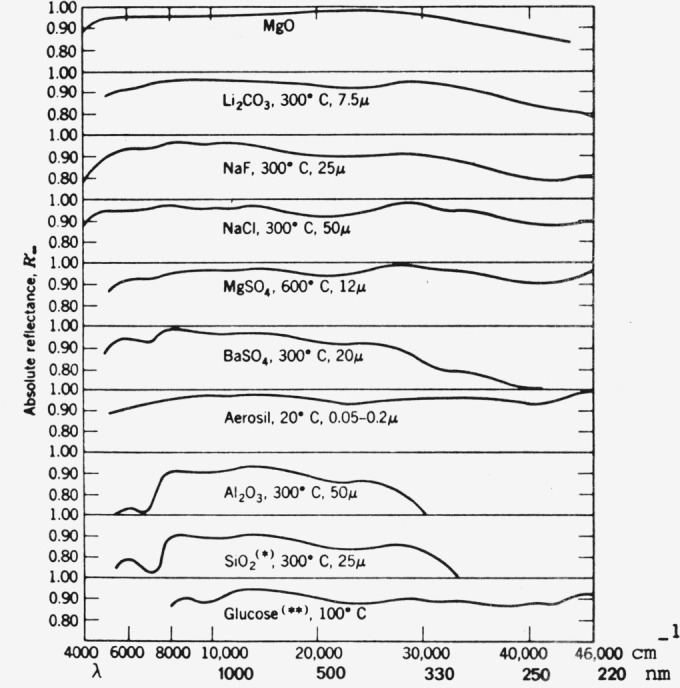
Absolute diffuse reflectance of several white standards as a function of wavelength. Measurements with reference to freshly prepared MgO. (Temperatures given are drying temperatures; µ=maximum grain size) [25].

A number of possible materials of fine powder texture have been investigated by Kortüm et al. [25], (see fig. 1). From this we can see that the condition of spectral nonselectivity is not ideally observed, particularly not in the lower UV-region. Of these MgO seems to have the best spectral properties and it has for a long time served as the most widely used standard. Its extreme sensitivity toward atmospheric conditions, UV-irradiation [27] and aging [14, 26] is well known. MgO-standards are also difficult to reproduce.

For this reason MgO has gradually been replaced by BaSO_4_ whose spectral properties are not as good (see fig. 1) but whose stability is superior.

The limited transportability of powder standards has prompted the development of glassy or ceramic standard materials such as Carrara, Didymium, Vitrolite etc., the latter being a white structural glass provided by NBS.

Since these materials have to have a matte surface (ideal diffusers), they are porous and therefore subject to contamination. Many of them have undesirable fluorescence and their spectral properties are inferior to the powders discussed particularly in the lower UV region < 250 nm.

The use of binders such as BaSO_4_, to render powder surfaces more stable, is another approach frequently used. Organic binders mostly have been used (polyvinyl alcohols) which seriously decreases the reflectance in the 200–300 nm region.

**Figure 2:**
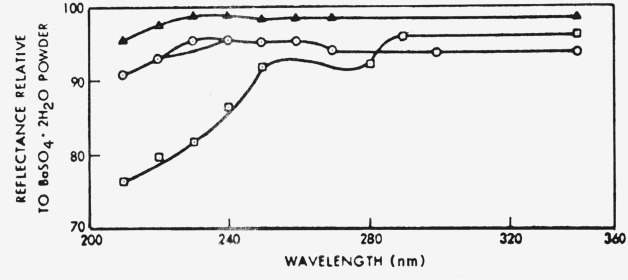
Reflectance spectra of barium sulphate coatings with different binders. ▲ K_2_SO_4_ binder; ⊙(NH_4_)SO_4_− K_2_SO_4_ binder; (NH_4_)_2_SO_4_ binder ⊡ Polyvinyl alcohol binder [28].

Schutt et al. [28] have suggested a number of inorganic binders which guarantee a similar mechanical stability but with significantly increased reflectivity in the 200–300 nm region (see fig. 2.) The same workers also claim improved aging and irradiation characteristics which should render this material suitable for transportable standards or as a highly reflecting coating in integration spheres.

From the foregoing discussion it is obvious that the development of better reference standards is still of prime necessity particularly for the UV region and much imagination and ingenuity will have to go into this research area.

As of 1969 the International Commission on Illumination (CIE) has replaced all previous primary standards by the “perfect reflecting diffuser,” possessing a reflectance of 100 percent. Since this can not be reached materially one can, beside the general improvement of standards, approach this problem from the instrumental side by basing the universally accepted reflectance scales on standardized instrumental designs. By relating such measurements on available standards to the “perfect reflecting diffuser” one obtains absolute reflectance values.

Instrument designs for the measurement of spectral radiance factors *β*(λ) have been discussed by several groups. A recent account and the description of an apparatus at the “Physikalisch-Technische Bundesanstalt,” Braunschweig, GFR, has been given by Erb [27].

This technique uses an integrating sphere with *d*/0 geometry and is based on the fact that the spectral radiance of the hemisphere irradiating the specimen could also be obtained if the “perfect reflecting diffuser” would be in place of the original standard.

Additional problems are encountered with the measurement and standardization of fluorescent surfaces, since for these the spectral distribution of the light source becomes another critical parameter. According to Berger and Strocka [29] artificial light sources coming as close as possible to the standard illuminant D 65 (xenon lamp) with regard to their irradiance distribution should be used.

The same group recommended the use of three standard samples for the assessment of fluorescent surfaces.

## IV. Investigation of Solid Surfaces

Diffuse reflectance spectroscopy can be a powerful tool for the physico-chemical study of surfaces. It can yield valuable complementary data to other surface techniques such as ESCA, regular microscopy, scanning electron microscopy etc. Kortüm has given an excellent survey of these possibilities in his book on reflectance spectroscopy [13]. Most of the work has been done on systems in the adsorbed state and much light has been shed on processes such as Lewis acid-base reactions, electron donor-acceptor complexation, redox reactions, photo-chemical reactions and others on solid surfaces. Adsorption phenomena have also been studied by Zeitlin et al., using this approach [14]. Use of reflectance techniques at elevated temperatures has been done by Wendlandt et al. [11, 12]. This group utilized either isothermal or dynamic (continuous measurement over a temperature range) high-temperature reflectance techniques to investigate the degradation, deaquation etc., of salts and metal complexes. These data were often used complementary to other thermoanalytical techniques, such as TGA and DTA.

**Figure 3:**
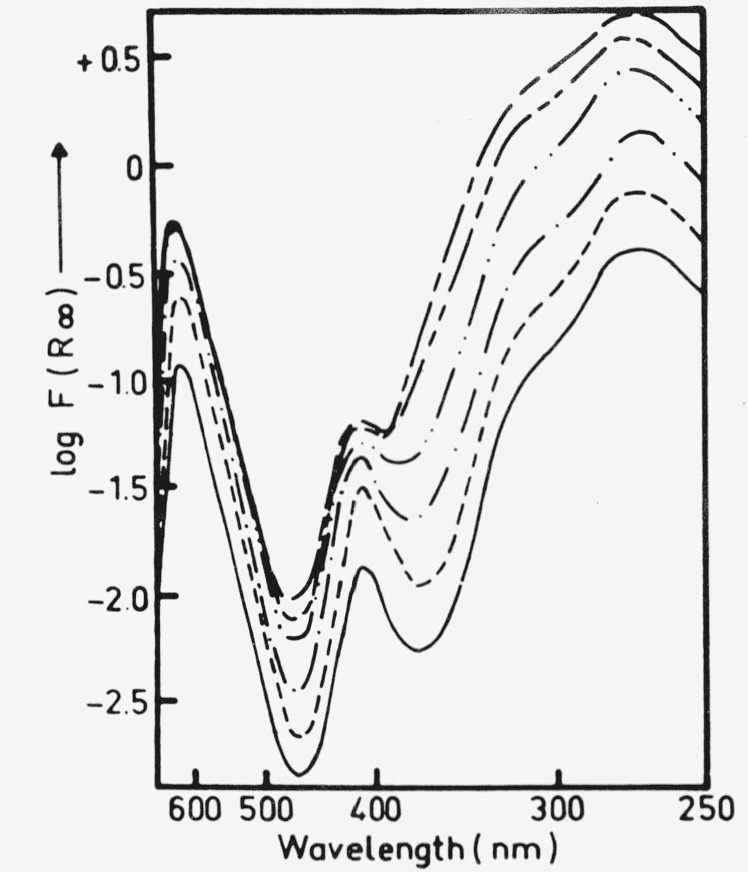
The dependence of the Kubelka-Munk functions of several bands of MGL on concentrations of MGL adsorbed on dry *NaCl* [30].

**Figure 4:**
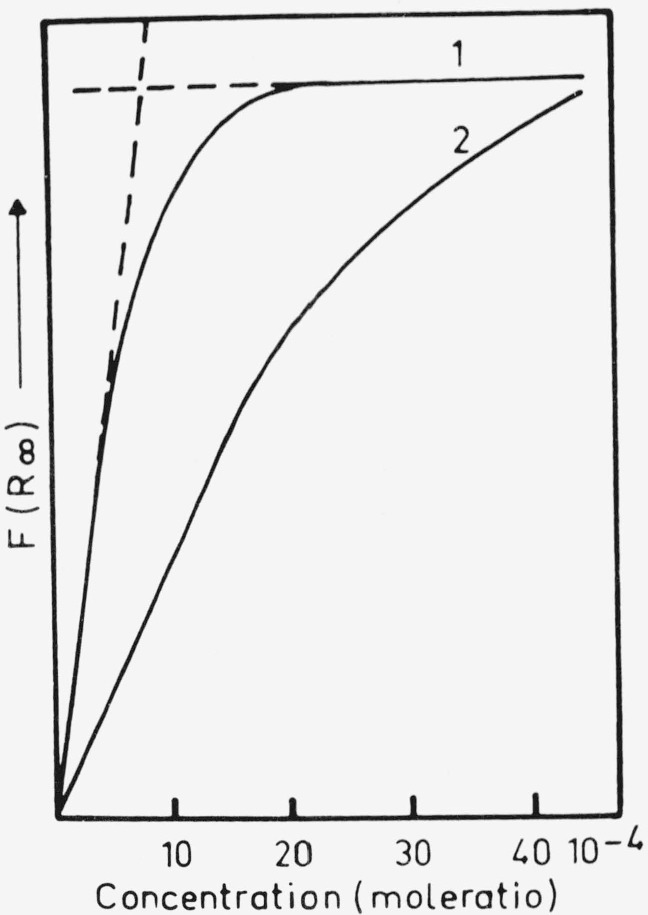
Adsorption isotherms of MGL adsorbed on dry *NaCl* [30].

Since chromatographic techniques often involve adsorption processes, the reflectance techniques can be adopted advantageously to preliminary investigations of actual chromatographic systems as one can see in the following example:

The reversible ring cleavage of compounds such as malachite green-*o*-carboxylic acid lactone (MGL) on active surfaces has been studied by Kortüm and Vogel [30] and Kortüm and Oelkrug [31]. A blue color develops upon adsorption of the colorless lactone on an activated adsorbent surface due to cleavage of the lactone ring. Since only chemisorption occuring in the first monomolecular layer can bring about this cleavage, the development of blue color will eventually reach a plateau at which point all adsorption sites have been covered. This phenomenon is demonstrated in figures 3 and 4. By extrapolation of the adsorption isotherm obtained for chemisorption, one can get a relative measure of the surface area of the adsorbent. The same phenomenon can also be observed on activated silica gels or alumina and offers an alternative to the BET-method for the determination of relative surface areas.

In modern high-pressure liquid chromatography adsorbents with pore sizes ranging from 60 A to 1000 Å and more and accordingly decreasing surface areas are now available. It would therefore be valuable to determine relative surface areas with molecules larger than N_2_ since, particularly in an adsorbent with small pore size, the nitrogen may reach active sites which are not necessarily available to the chromatographed organic compounds. The availability of such data facilitates the choice of the proper adsorbents with regard to loading capacity and retention properties.

## V. Calibration

### A. Calibration Functions

The Kubelka-Munk function [32, 33] for lightscattering, infinitely thick media, is most widely used for investigations of a quantitative analytical nature by diffuse reflectance spectroscopy. Many different types of calibration functions have been proposed for the in situ evaluation of thin-layer or other open, flat-bed chromatograms by UV or visible reflectance spectroscopy [14, 19]. Most of the functions were modifications of the Beer-Lambert or the KubelkaMunk laws; others were purely empirical. All of them claim reproducible linearity over various concentration regions even though the limitations are quite serious. The Beer-Lambert function gives reasonable linearity at very low concentrations; the KubelkaMunk function works at medium concentration ranges but fails at lower and higher concentrations. The limitations of the Kubelka-Munk theory are quite obvious for chromatographic systems, since except for monochromaticity of the irradiated light, other conditions such as infinite layer thickness, homogenous distribution of absorbing material and nonabsorbing support are in most cases not fulfilled.

**Figure 5:**
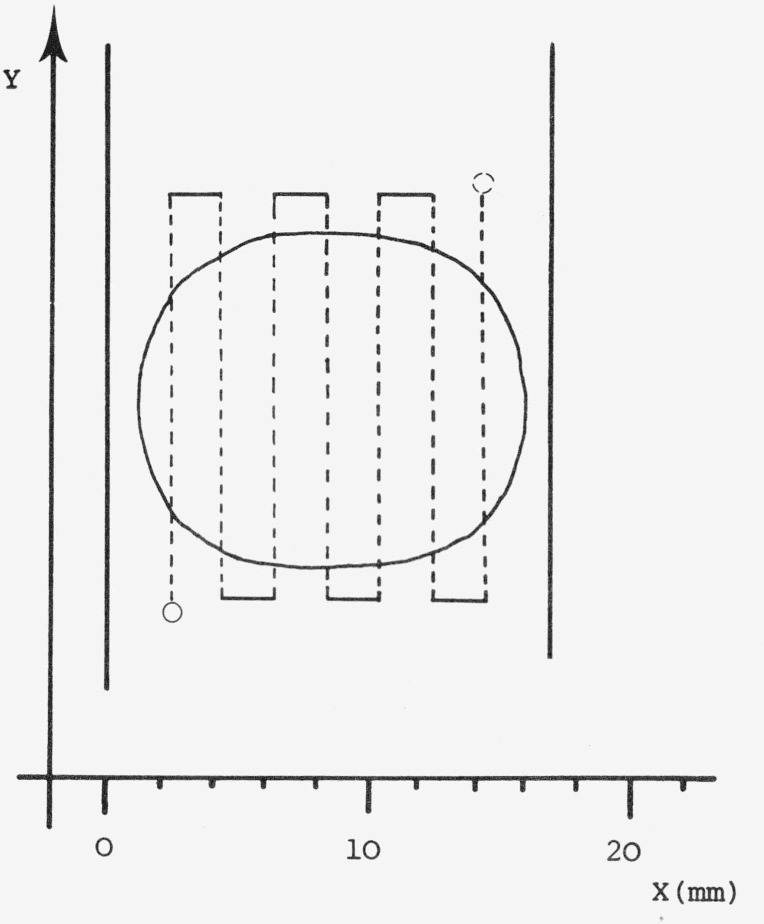
Schematic representation of the scanning pattern employed for the two-dimensional integration principle [35].

The inhomogeneity caused by tailing or other deformation of chromatographic zones has been countered by some workers by scanning chromatographic zones with a small spot of monochromatic light in a zig-zag fashion (flying spot principle [34]). Equal success, but with a technically simpler approach, has been reported by Treiber et al. [35], with a two-dimensional scanning approach (fig. 5). Such a scanning device is now commercially available. The same group [36] has proposed a combination of the two spectroscopic laws (Lambert-Beer and Kubelka-Munk; as shown below) in order to extend the linearity of the calibration range.

$$K_{x}\cdot C=K_{t}\cdot \ln\frac{I_{0}}{I_{x}}\text{Lambert-Beer}$$ (1)

$$\begin{array}{r} K_{x}\cdot C=K_{r}\cdot \frac{{\left(1-\frac{I_{x}}{I_{0}}\right)}^{2}}{2\cdot \frac{I_{x}}{I_{0}}}=\frac{{\left(1-R_{\infty }\right)}^{2}}{2\cdot R_{\infty }}=k\cdot \epsilon \cdot C\\ \text{Kubelka-Munk} \end{array}$$ (2)

by combining 1 and 2 one obtains:

$$K_{x}\cdot C=K_{r}\cdot \frac{{\left(1-\frac{I_{x}}{I_{0}}\right)}^{2}}{2\cdot \frac{I_{x}}{I_{0}}}+K_{T}\cdot \ln\frac{I_{0}}{I_{x}}$$ (3)

A simple transformation: leads to

$$K_{x}\cdot C=K_{R}\cdot \left(\frac{I_{0}}{I_{x}}+\frac{I_{x}}{I_{0}}-2\right)+K_{T}\cdot \ln\frac{I_{0}}{I_{x}}$$ (4)

*K_x_*: =constant depending on the substance chromatographed
*K_R_*(*K_r_*), *K_T_*(*K_t_*): =constants depending on the properties of the adsorbent layer (determined empirically)
*k*: =constant depending on the adsorbent layer
ϵ: =molar absorbance of the substance chromatographed
*C*: =concentration of the substance chromatographed in weight per surface unit
*I_x_*: =intensity of the light leaving the sample
*I*_0_: =constant, maximal light intensity on the adsorbent layer free from any substance chromatographed.

A comparison of the *K–M*–function with the combined function eq (4) for sulforidazine scanned automatically by the two-dimensional technique at 275 nm is shown in table I with some statistically computed values.

A significant improvement can be noted particularly for the lower concentration region (0–2 *µ*g).

**Figure 6:**
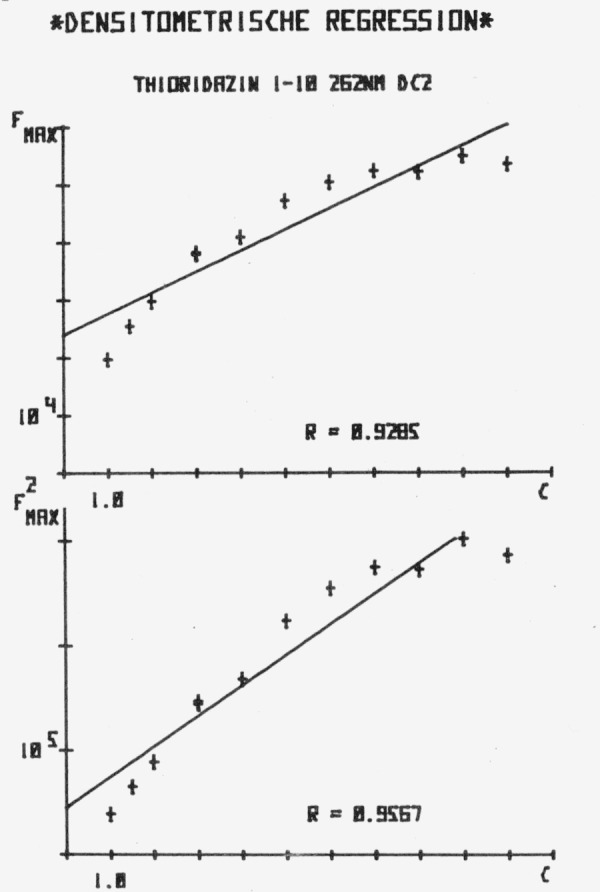
Calibration curves peak area (*F*) and peak area square (*F^2^*) for thioridazine in the concentration range 1–10 µg/spot at λ=262 nm.

Our own work [45] confirms that significant improvements can be obtained using the Treiberfunction in comparison to plotting peak area or peak area square (*F*_max_ or $F_{\max}^{2}$ in fig. 6). The study was carried out with thioridazine as a test substance on Merck SiO_2_ commercial plates. The measurements were carried out with a Zeiss-Chromatogram Spectrophotometer PMQ II, with automatic scanning unit.

For the data aquisition a combination with Infotronics Integrator CRS 208, W+W-recorder, Teletype ARS 33 and Hewlett-Packard HP-Calculator 9830 with plotter was used.

The following figures are direct plotter read-outs. Measurements were made at the two possible absorption maxima for thioridazine at 262 nm and at 315 nm.

**Figure 7:**
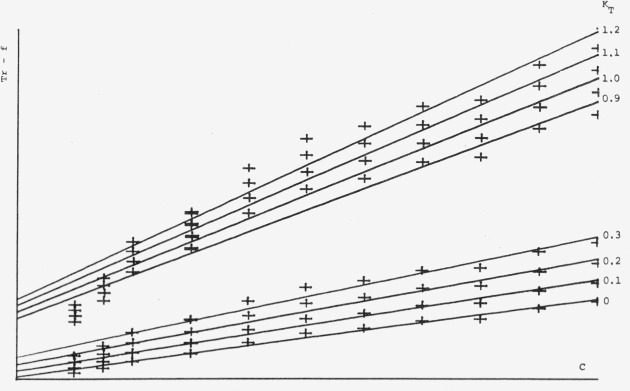
Treiber-funclions (*Tr.-f*) with varying *K_T_*-values. Conditions as in figure 6.

**Figure 8:**
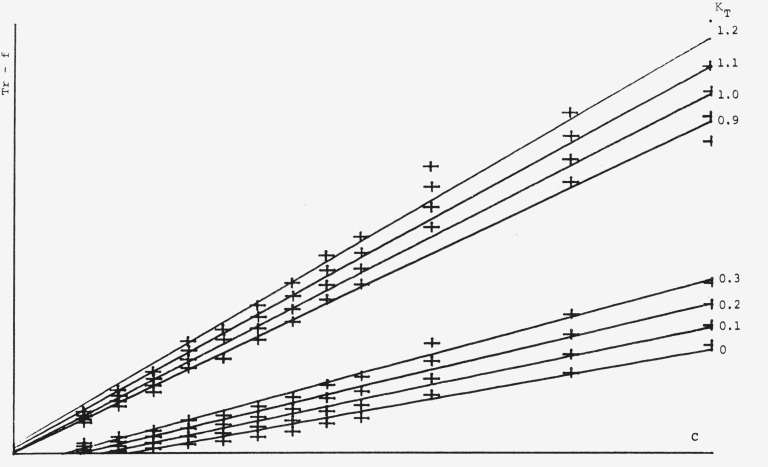
Treiber-functions (*Tr.-f*) with varying *K_T_*-values at 262 nm for a concentration range 0.2–2 μg/spot.

Figure 6 shows the relatively poor correlations obtained with the commonly used function *A* (*F*_max_) and *A*^2^ ( $F_{\max}^{2}$) over a concentration range of 1–10 *µg/*spot. For calibration plots using the Treiber-function, *K_R_* in eq (4) has been kept constant at 1 and *K_T_* has been varied as can be seen in figure 7. The best calibration plot going through the point of origin has been obtained for *K_T_*=0, which means that the Beer-Lambert-term dropped out and that the system behaves essentially according to the Kubelka-Munk function. By studying the same system at a different concentration range (0.2–2 *µ*g/spot) we can see in figure 8 that the Treiber-function is quite concentration dependent, now the optimum value for *K_T_* is 0.9 to 1.1 This means that the influence of the transmission effect (Beer-Lambert-function) is about equal to the reflectance effect (*K-M*-function), which is somewhat astonishing since at 262 nm one would not expect any energy to be transmitted through the plate.

**Figure 9:**
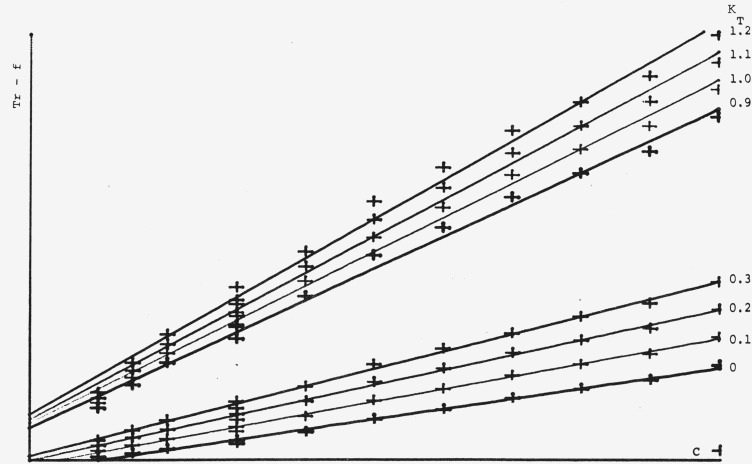
Treiber-functions (*Tr-.f*) with varying *K_T_*-values at 315 nm for a concentration range 1–10 µg/spot.

The Treiber-function is also wavelength dependent. This can be observed by comparing figure 9 to figure 7 where measurements have been made at 315 and 262 nm respectively at the same concentration range (1–10 *µ*g/spot). The optimum *K_T_*-value in figure 9 would be around 0.3 which means that a portion of the light is being transmitted. This is to be expected at 315 nm. The reason for the *λ*-dependence of the Treiber-function is easily explained, but at the moment we are unable to give a satisfactory reason for the concentration dependence. Treiber has not observed or at least not reported on these very important effects.

The above experiments demonstrate also that it is now possible to automate completely the entire densitometric measurement and data evaluation steps. This should enhance the usefulness of densitometric methods for routine analysis.

### B. Control of Linearity

No matter which functions are utilized, one of the major requirements for a good quantitative procedure is a proper linear working range. This should be controlled by consistently checking the correlation coefficients of the calibration curves. A simple approach to this end which is frequently used in our control laboratories is described below.

For the purpose of saving space for the application of unknowns, the system was restricted to 3 standard concentrations, hence only three fixed points determine the calibration curve and therefore even highly significant correlation coefficients do not yield much information.

**Figure 10:**
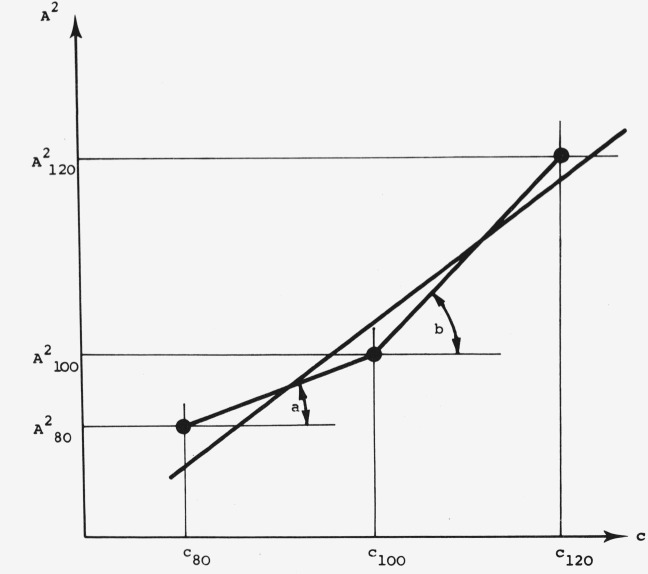
A schematic calibration curve depicting the deviation from ideal correlation. *c*_80_, *c*_100_, *c*_120_: concentration of the standard solutions with 80 percent, 100 percent, 120 percent of the expected concentration of unknown.*A*^2^_80_, *A*^2^_100_, *A*^2^_120_: squared values of the measured peak areas of the spots with *c*_80_, *c*_100_, *c*_120_

In order to permit meaningful control of the linearity of our working concentration range and to have an absolute value of slope deviations from linearity a more realistic approximation has been proposed (see fig. 10).

$$A^{2}=mc+n$$ (5)

$$A_{1}^{2}=ac+n_{1}$$ (5a)

$$A_{2}^{2}=bc+n_{2}$$ (5b)

$$a=\frac{A_{100}^{2}-A_{80}^{2}}{c_{100}-c_{80}}$$ (6)

$$b=\frac{A_{120}^{2}-A_{100}^{2}}{c_{120}-c_{100}}$$ (7)

The total regression of the 3 calibration points can be separated into two segments. As can be seen in figure 10, a deviation from ideal correlation has its most serious effect when the concentration of the unknown is closest to the ideal 100 percent standard concentration; in other words, the largest error could be expected for the best samples.

The magnitude of this poor correlation can be seen by comparing the slopes *a* and *b* respectively of the two segments (fig. 10): According to this, the slope difference *b–a* is a measure of the calibration quality. With this quality factor, it is also possible to screen for the best linearity range. If *b—a* is consistently positive or negative, we are definitely in a nonlinear range and another concentration region has to be chosen.

This systematic error cannot be seen by calculating the correlation coefficient.

**Figure 11:**
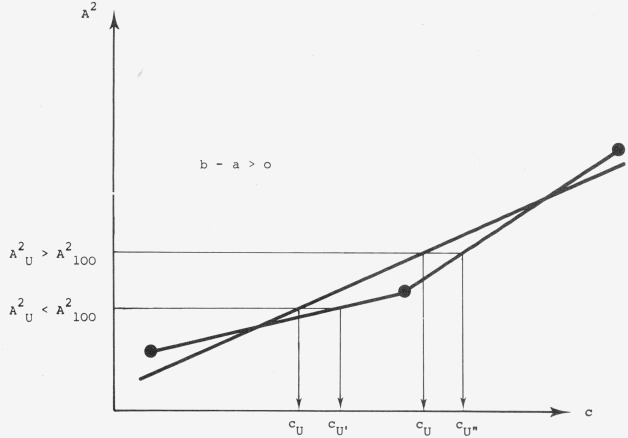
A schematic presentation of the errors that can he expected due to calibration errors.

After determination of the slope of each segment, one can compute the error of the analytical result, caused by the calibration error (see fig. 11).

*c_U_*: =concentration of unknown
*c_U_*_exp_.: =expected concentration of unknown (true concentration)

$$\frac{c_{U}}{c_{U}{\;}_{\exp.}}\cdot 100=U[\%]$$ (8)

$$\frac{c^{\prime }{\;}_{U}}{c_{U\exp.}}\cdot 100=U^{\prime }[\%]$$ (9)

$$\frac{c^{\prime \prime }{\;}_{U}}{c_{U\exp.}}\cdot 100=U^{\prime \prime }[\%]$$ (10)

From eqs 8–9 one derives the definitions for analytical errors Δ*U*

$$\Delta U=U^{\prime }-U[\%]$$ (11)

$$\Delta U=U^{\prime \prime }-U[\%]$$ (12)

consequently we have

for condition *b–a*>O:Δ*U*>O
for condition *b–a*<O:Δ*U*<O.

The term Δ*U* according to eqs 11 and 12 should always be considered for the computation of analytical results.

Chromato-plates which yield a Δ*U* higher than 2*σ* of the method should be rejected since for a 95 percent probability the analytical results have to be within $\bar{U}\pm 2\sigma$.

### C. Transferability of Calibration Functions

The difficulties of external or transferable calibration for the reflectance spectroscopic evaluation of thin-layer chromatograms are well known [14]. Usually each plate has to carry its proper set of standards. A quantitative method with transferable calibration curves by means of an internal standard has been described by Klaus [37, 38], but its general applicability is somewhat questionable. Our efforts were also directed towards achieving a maximum of external transferable information to enhance the analytical capacity of the method [39].

**Figure 12:**
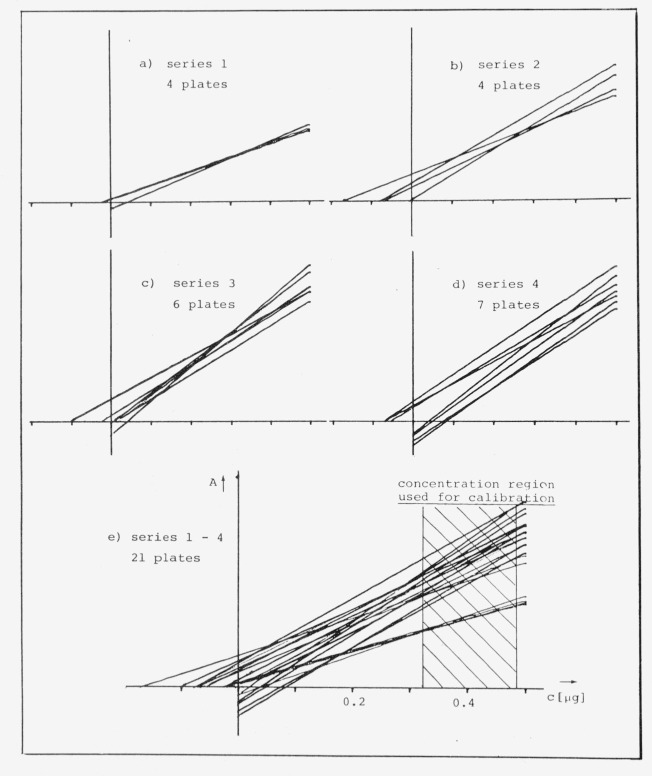
Calibration plots *A=a+bc* for four different series of compound *x* [39].

The usual picture for a set of calibration curves from different plates (see fig. 12) is a large variation of slopes for a given concentration range and the curves do not usually pass through the origin. As a result it is impossible to have a complete external calibration. At least two measured points are required to determine the coefficients for slope *b* and the ordinate intercept *a* (*A=a+bc*).

If one attempts to clear the tangle of calibration curves in figure 12 by sorting them out according to experimental series, one can see that they differ more from series to series than within the same series (figs 12a–12d). It is also apparent that they vary less in slopes *b* than in intercept *a.*

On the basis of these observations, we have tried to use the slope *b* for external calibration.

This was done by two different approaches:

#### 1. Direct Transfer of the Mean Slope-Value

$\bar{b}$ is computed from the slope values of several plates

$$\begin{array}{l} A=a_{1}+b_{1}\cdot C\ldots \ldots \;A=a_{n}+b_{n}\cdot c\\ \bar{b}=\frac{\sum b_{i}}{n}(b_{i}\;\text{individual slope value}) \end{array}$$ (13)

and is selected as the direct transfer factor. The individual ordinate intercept *a_i_* for each plate is then computed from a single calibration value as shown below. (*A_x_, c_x_*: peak area and concentration of the standard).

**Figure:**
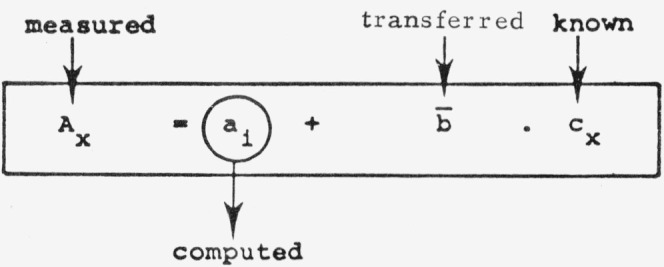

#### 2. Transfer of a Relative Slope-Value

This approach was selected in order to compensate for the fluctuation of mean slope values $\bar{b}$between the different series (fig. 12).

Value *b* for each plate is divided by a reference value *A_id_* which is ideally the mean value of the range of calibration.

$$\begin{array}{l} A_{id}=\frac{\sum A_{ST_{i}}}{n}\\ A_{ST_{i}}=\text{individual peak area of standard}. \end{array}$$ (14)

With three standards used as is usually the case for our routine methods this would be

$$A_{id}=\frac{A_{ST1}+A_{ST2}+A_{ST3}}{3}$$ (15)

The mean relative slope value $\bar{b_{\text{rel}}}$ is then computed for several plates

$$\begin{array}{l} b_{{\text{rel}}_{1}}=\frac{b_{1}}{A_{id_{1}}}\ldots \ldots b_{{\text{rel}}_{n}}=\frac{b_{n}}{A_{id_{n}}}\\ \to \bar{b_{\text{rel}}}=\frac{\sum b_{{\text{rel}}_{i}}}{n}(b_{{\text{rel}}_{i}}:\text{individual rel}.\;\text{slope value}). \end{array}$$ (16)

Slope *b_i_* of the individual calibration curve on each plate is calculated by back-multiplication of the measured value *A_x_* of a single calibration spot with $\bar{b_{\text{rel}}}$ (see scheme below). The individual ordinate intercept *a_i_* is then computed.

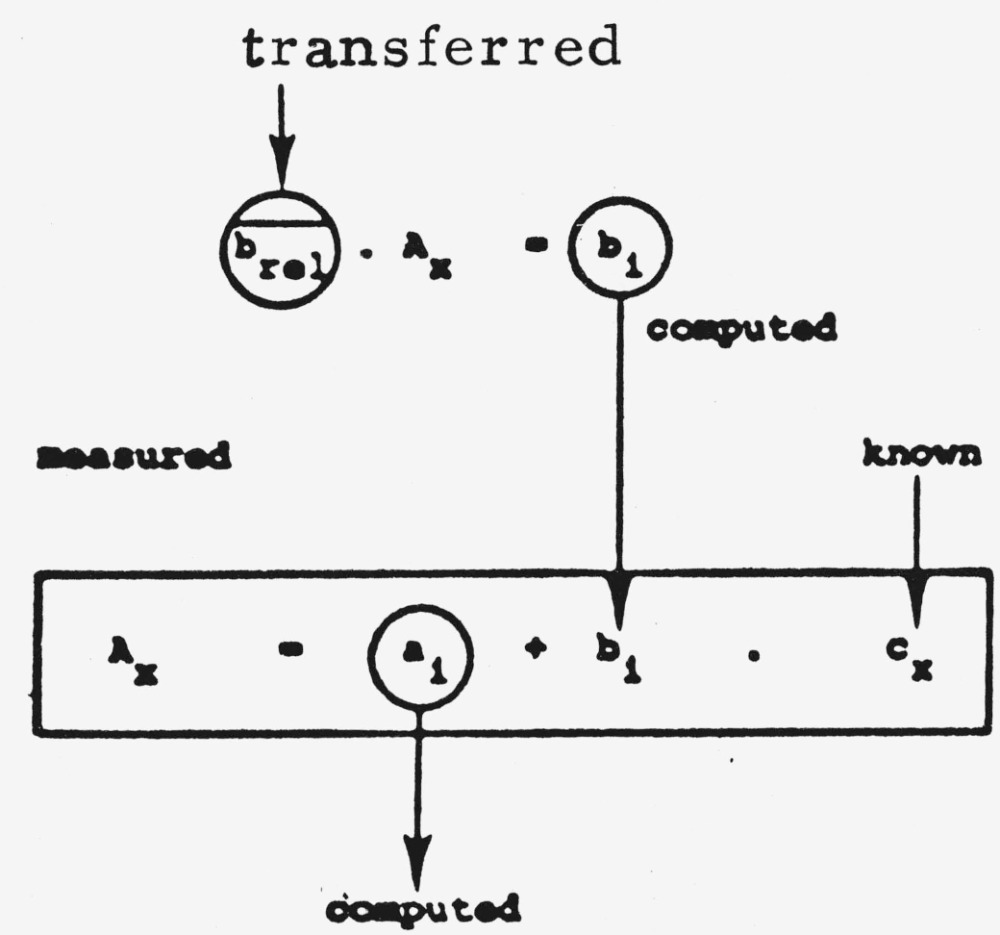
(17)

A comparison of these two techniques of transferred calibration was carried out with a drug substance for which multiple analyses were carried out (see also fig. 12).

The results are shown in table II. As is to be expected no significant improvement is observed between direct and relative transfer when the four separate series are considered. For the total series, however, the mean error for the direct transfer approach is twice as high.

The mean error Δ*U*_rel_ is defined as

$$|\Delta U_{\text{rel}}|=\frac{U_{\text{transf}.\;\text{calibr}.}-U_{\text{individual calibr}.}}{U_{\text{individual calibr}.}}\cdot 100.$$ (18)

Thus, table II also shows, that transferred calibration techniques are practically equivalent to individual calibration on each plate. Five analysis can be carried out per plate which represents an increase in capacity of 60 percent. The method is applicable to UV, visible and fluorescence measurements by reflectance techniques and also after utilizing dipping or spraying procedures.

The only limitation is that the test series is from the same concentration region and of course that they fall into a linear calibration range.

### D. Multicomponent Systems

If this condition of linearity is adhered to (no matter which function is used) then it is also possible, analogous to regular spectrophotometric principles, to carry out differential reflectance measurements with highly or weakly absorbing compounds [40] or to evaluate multicomponent systems [41].

The latter has been demonstrated with dye mixtures on a silica gel substrate, and the KubelkaMunk-function was used eq (2) as a linear relationship in the concentration range studied.

For a powder mixture containing *n* light-absorbing components whose reflectance functions are additive, the Kubelka-Munk function *F* (*R*_∞_) can be adapted for simultaneous analysis. The function of the total reflectance *R*_∞_*_T_* of the mixture at some wavelength *i* may be represented as the sum of all individual reflectance functions.

$$F{\left(R_{\infty T}\right)}_{i}=\sum_{j=1}^{n}\tau_{ij}C_{j}$$ (19)

where *j* refers to components and *τ* is the slope of the Kubelka-Munk plot of *F*(*R*_∞_) versus *C.*

Equation (19) can be written in a more explicit manner by writing as many equations as there are components in the mixture.

$$F{\left(R_{\infty T}\right)}_{1}=\tau_{11}C_{1}+\tau_{12}C_{2}+\ldots \tau_{1n}C_{n}$$ (20)

$$F{\left(R_{\infty T}\right)}_{2}=\tau_{21}C_{1}+\tau_{22}C_{2}+\ldots \tau_{2n}C_{n}.$$ (21)

**Figure 13:**
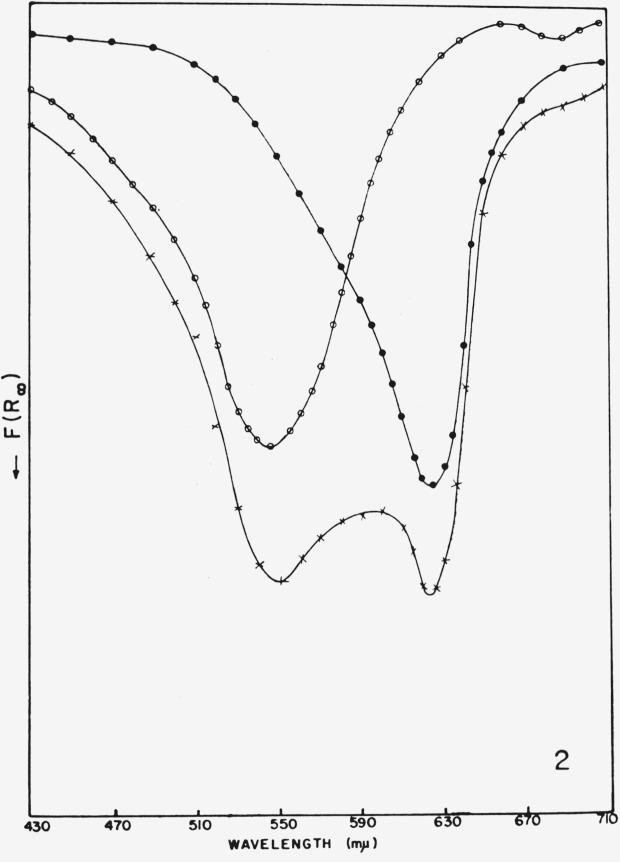
Reflectance spectra of ○, Fuchsin; ●, Brilliant green; *x*, a mixture of the two [41].

**Figure 14:**
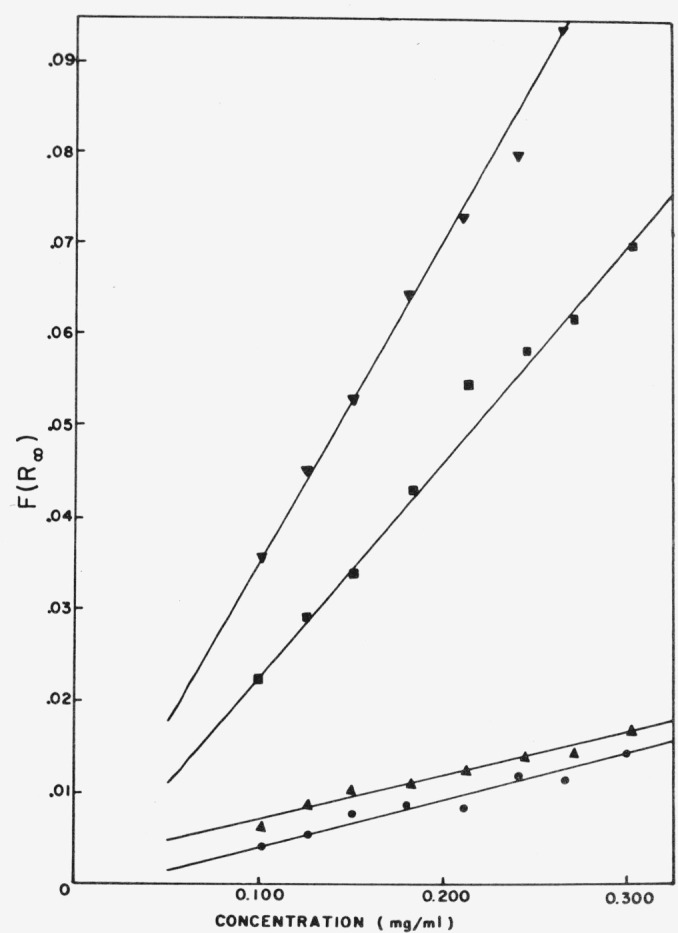
Kubelka-Munk plots for ▼, Brilliant green at 626 nm ▲, Fuchsin at 626 nm; ● Brilliant green at 545 nm and ■, Fuchsin at 545 nm [41].

The additivity of the *F*(*R*_∞_) – values is shown in figure 13 for a mixture of Fuchsin and Brilliant green. From the corresponding calibration curves (fig. 14) the slopes are determined and utilized in eqs. (19–21) for a computation of the individual concentrations.

The precision, computed as relative standard deviation for four sets of four samples, was ±2.4 percent and the deviation from the true value (accuracy) was 2.1 percent for Fuchsin and 3.1 percent for Brilliant green.

The same principle is also applicable in the UV-region. Mixtures of rutile and anatase [42] have been studied. Similar applications in the color industry and pharmaceutical industry have also been discussed [14].

## VI. Applications in Chromatography

Applications have been discussed in recent books [14, 15, 19] and the latest instrument developments have greatly enhanced the use of diffuse reflectance spectroscopy in this area. I do not feel that there is a need in this paper to go into further detail. I would just like to mention one recent application area mainly UV-reflectance spectroscopic measurements of chromatographic zones on silica gel in the wavelength range of 180–210 nm. U. Hexel [43] has shown that with the necessary precautions such as nitrogen purge below 195 nm (ultrapure N_2_ below 190 nm), the use of good optics (to eliminate stray-light effects), and a special deuterium lamp and window (all items commercially available), it is possible to obtain quantitative results in this spectral region. This is somewhat surprising since it is well known that silica gel starts absorbing strongly below 280 nm (see also fig. 1). However, the determination of the absolute reflectance of silica gel in this spectral region is difficult since good UV-reflectance standards are lacking.

**Figure 15:**
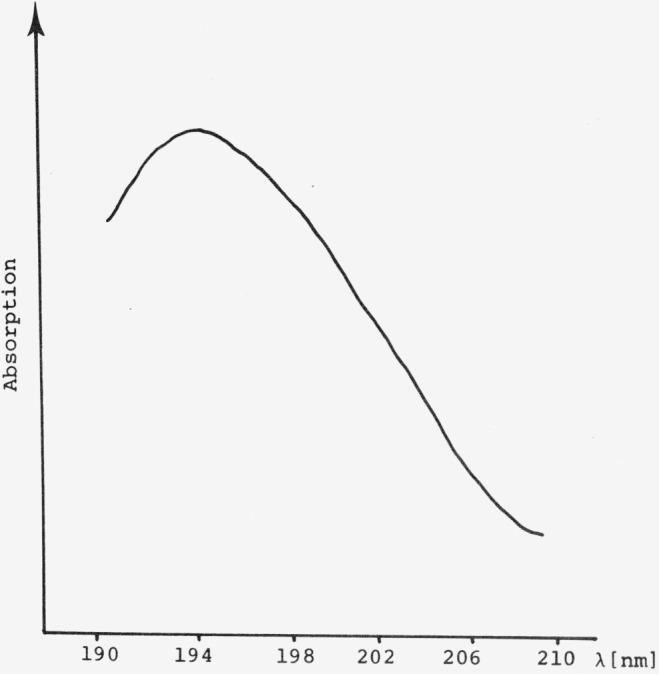
Absorption spectrum of trioleine standard measured by diffuse reflection [43].

**Figure 16:**
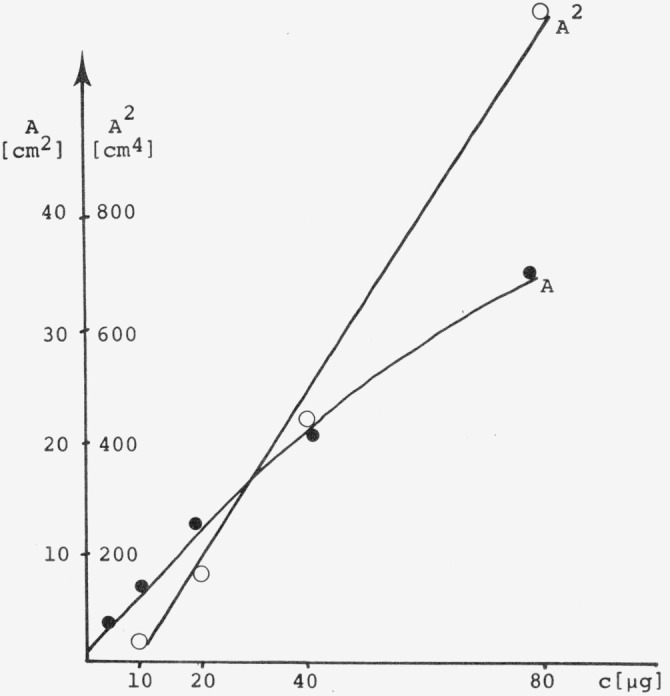
Calibration curves for the trioleine standard measured at 196 nm [43].

The absorption spectrum and calibration curves of a trioleine compound [43] (triglyceride) are shown in figures 15 and 16. Measurements were done with the Zeiss Chromatogram spectrophotometer equipped with a suprasil window and a deuterium lamp H 30 DS. No nitrogen purge was necessary at the working wavelength λ196 nm. A precision of ± 2 percent rel. S.D. is possible.

This approach has much merit for the investigation of compounds with *C* = *C, C* = O or C *= N* bonding and otherwise poor chromophores.

Actual groups of compounds tested to this date are caprolactams (*λ*=194 nm) [43], atropine sulfate (*λ*=195 nm) [43], Lipids (*λ*=196 – 210 nm) [44] and some peptides (*λ*=200 – 220 nm) [45].

## VII. Conclusion

In conclusion one can say that diffuse reflectance spectroscopy is by no means a forgotten or obsolete technique. It is adopted heavily in highly specialized areas such as color measurement and chromatographic techniques. Automation and computer usage play an important role in both areas and the instrument technology is at a good level.

Some helpful innovations have been made on calibration techniques which can make quantitative diffuse reflectance spectroscopy competitive in some cases with transmission techniques.

The problem of standardization is very much with us with regard to finding better reference materials and better standardization of instruments. A great deal of effort and imagination will have to go into this areas for genuine progress.

## Figures and Tables

**Table I:** A statistical comparison of calibration curves for sulforidazine

	Range *μ*g	Slope	Correlation	Relative st. dev.

K-M-				
function:	0–10	1079	0. 99778	25. 5 (± 2.4 %)
	0–2	733	. 97005	91. 7 (±12. 5 %)
	2–10	1154	. 99941	22. 9 (± 2. 0 %)
Combined				
function:	0–10	2088	0. 99991	9. 6 (± 0.46%)
	0– 2	2088	. 99876	52. 1 (± 2.5 %)
	2–10	2069	. 99988	18. 3 (± 0. 88%)

**Table II:** Mean values of the relative errors of analyses of compound *x* corresponding to figure 12

|Δ*U*_rel_|-Values after transfered calibration		
		
Transferred value	4 Separated groups	Total series
		
$\bar{b}$	0.9%	1.2%
$\bar{b_{\text{rel}}}$	0.6%	0.6%
